# Sustainable Extraction of Mangiferin From Mango Leaves Using Taguchi and Box‐Behnken Optimization: Biochemical Characterization and Antioxidant Profiling

**DOI:** 10.1002/jssc.70500

**Published:** 2026-08-20

**Authors:** Astrid Corrales, Vitor de Pádua Alves, Júlia Naelly Machado Silva, Felipe Rebello Lourenço, Massuo Jorge Kato, André Moreni Lopes

**Affiliations:** ^1^ Bioseparations and Nanoformulations Laboratory (BIOSEP NANOLAB) Department of Biotechnology Lorena School of Engineering University of São Paulo (DEBIQ, EEL/USP) Lorena Brazil; ^2^ Lorena Technical School (COTEL) – Prof. Nelson Pesciotta – Lorena School of Engineering University of São Paulo Lorena Brazil; ^3^ Natural Products Chemistry Laboratory Institute of Chemistry University of São Paulo (LQPN, IQ/USP) São Paulo Brazil; ^4^ Department of Faculty of Pharmaceutical Sciences LabMEQ ‐ Laboratory of Metrology, Examinology, and Quality by Design University of São Paulo São Paulo Brazil

**Keywords:** ethanol‐water systems, extracting optimization, green extraction technologies, natural xanthones, radical scavenging, sustainable bioprocessing

## Abstract

Mangiferin (MGF), a bioactive C‐glucosylxanthone predominantly found in *Mangifera indica* L., has attracted increasing interest due to its recognized antioxidant and pharmacological properties. In this study, an aqueous ethanolic maceration process was systematically optimized for MGF recovery from mango plant biomass using a Taguchi experimental design followed by Box‐Behnken response surface methodology. The optimized conditions of 39.9°C, 8 h, and 80% v/v ethanol yielded 41.60 mg/g dry weight of MGF, confirming the robustness of the predictive model. Biochemical profiling of the optimized extract revealed 4.36 mg gallic acid equivalents/g dry weight of total phenolics, 73.12 mg/g of reducing sugars, and low protein content, indicating a phytochemical matrix enriched in glycosylated phenolics. Antioxidant evaluation by the 2,2‐diphenyl‐1‐picrylhydrazyl radical scavenging assay demonstrated 87.74% radical inhibition at 400 µg/mL and a 50% inhibitory concentration of 225 µg/mL, reflecting strong radical scavenging capacity. Greenness assessment using the Analytical GREEnness metric approach software yielded a score of 0.72, validating the environmental compatibility of the proposed method. The results demonstrate that statistically guided aqueous ethanolic maceration represents an efficient, scalable, and sustainable platform for the recovery of MGF‐rich extracts with potential pharmaceutical, nutraceutical, and cosmetic applications.

## Graphical abstract

A sustainable aqueous‐ethanolic maceration process, sequentially optimized using Taguchi and Box‐Behnken designs, enabled efficient mangiferin recovery from *M. indica* leaves. The optimized extract exhibited high antioxidant activity and a favorable Analytical GREEnness score, demonstrating its potential as a scalable and environmentally responsible extraction platform.

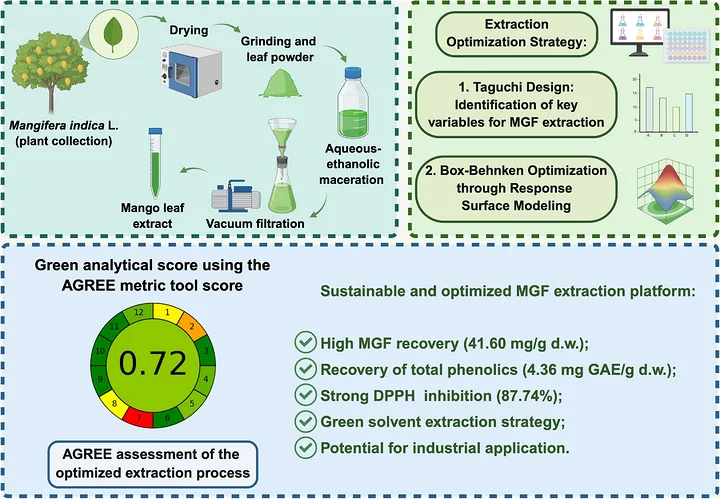

## 1 Introduction

Throughout human history, plant‐derived biomolecules have played a central role not only in nutrition but also in the prevention and treatment of numerous diseases. Among these bioactive compounds, mangiferin (MGF), a naturally occurring C‐glucosylxanthone predominantly found in mango trees (*Mangifera indica* L.), has attracted growing scientific interest due to its wide spectrum of therapeutic properties. The mango tree, belonging to the Anacardiaceae family, has been extensively cultivated in tropical and subtropical regions and is valued not only for its fruit but also for the remarkable phytochemical richness of its bark, seeds, leaves, and roots. These anatomical parts contain a diverse array of compounds, including flavonoids, phenolic acids, tannins, and terpenoids, that contribute to their traditional and contemporary medicinal applications [1, 2, 3].

**FIGURE 1:**
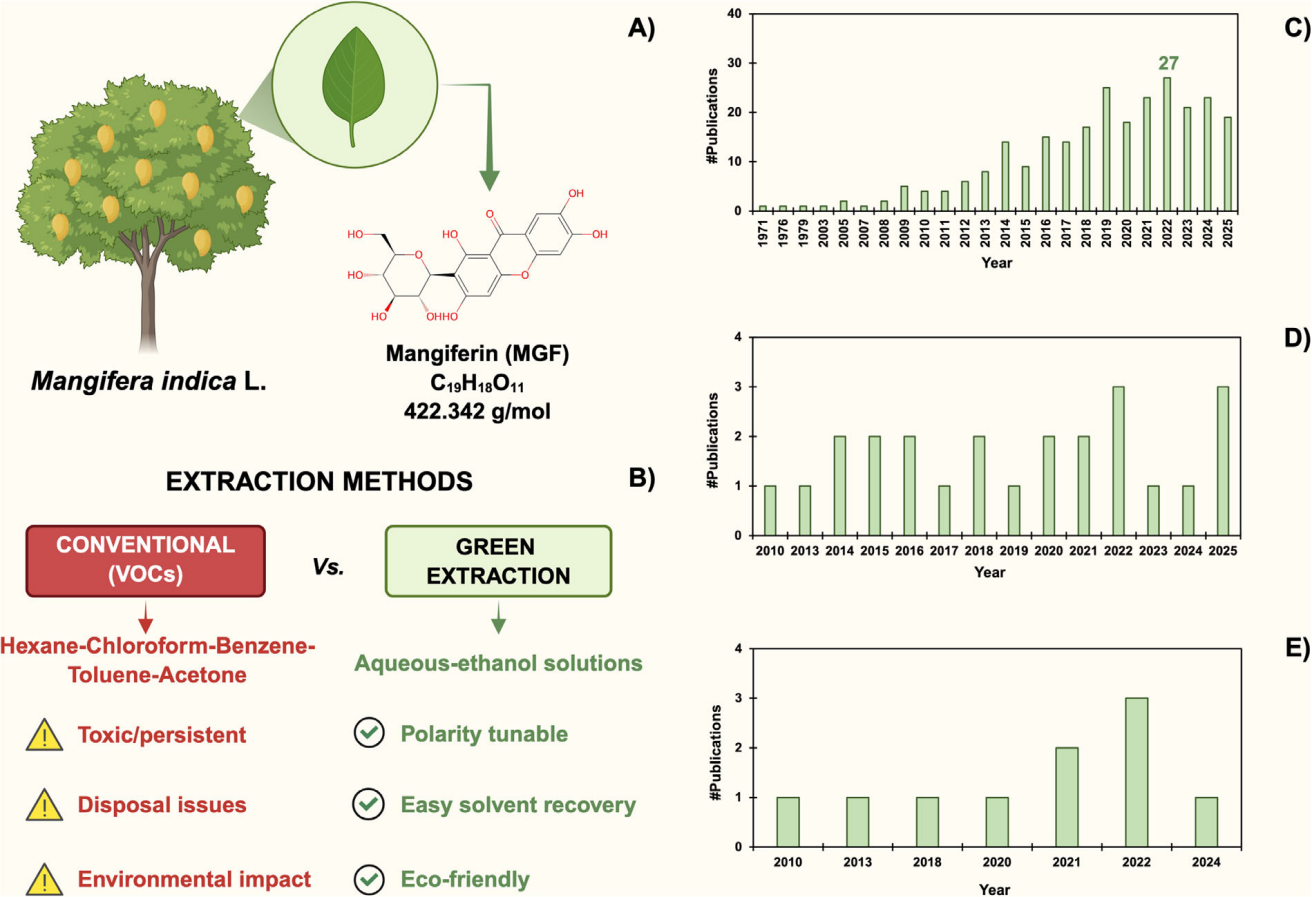
Overview of mangiferin (MGF) research landscape. Botanical representation of *M. indica* L., highlighting leaves and fruits as primary anatomical sources of MGF and the chemical structure of MGF (1,3,6,7‐tetrahydroxyxanthone‐C2‐β‐D‐glucoside), the predominant C‐glucosylxanthone in mango leaves (A). Comparison between conventional extraction techniques employing volatile organic compounds (VOCs) and sustainable green extraction approaches based on aqueous ethanol solutions (B). Bibliometric trends obtained from Web of Science (December 12, 2025) illustrating the evolution of scientific publications over time using the keyword sets: “mangiferin” and “extraction” (C), “mangiferin” and “conventional extraction” (D), and “mangiferin” and “ethanol‐water” (E).

MGF, chemically described as 1,3,6,7‐tetrahydroxyxanthone‐C2‐β‐D‐glucoside (C_19_H_18_O_11_; *M*_W_ 422.34 g/mol, Figure 1A) (ChemSpider [4]), exhibits a molecular structure optimized for hydrogen donation and electron transfer, supporting its potent free radical scavenging properties. This antioxidant capacity is complemented by its ability to modulate endogenous enzymatic defenses such as superoxide dismutase, catalase, and glutathione peroxidase, reinforcing cellular protection, redox homeostasis, and overall immune function [5, 6, 7, 8, 9]. In addition to its antioxidant role, MGF displays antimicrobial, antiviral, anti‐inflammatory, hepatoprotective, hypoglycemic, and immunomodulatory activities, further elevating its relevance for pharmaceutical, nutraceutical, and cosmetic applications [8, 10, 11, 12]. Emerging evidence also points to its capacity to modulate pathways involved in apoptosis and cell proliferation, supporting its potential contribution to anticancer strategies.

Despite the extensive literature describing the biological relevance of MGF, important challenges remain concerning its efficient extraction, purification, and stabilization. Among conventional solid‐liquid extraction approaches, maceration is one of the most widely employed techniques due to its simplicity, low energy demand, minimal equipment requirements, suitability for processing plant biomass, and high operational scalability [13, 14, 15, 16, 17]. However, traditional maceration has mostly been based on the use of hexane, chloroform, and ethyl acetate, among others, which, although capable of achieving good extraction performance, introduce major limitations. These solvents raise concerns related to toxicity, environmental persistence, flammability, specialized waste management, and strict regulatory constraints, especially for pharmaceutical, nutraceutical, and cosmetic applications [13, 18, 19, 20, 21, 22]. Moreover, studies employing such solvents often report inconsistent extraction yields due to the absence of systematic optimization of key variables such as solvent composition, temperature, and extraction duration. Such methodological inconsistencies compromise reproducibility, limit scalability, and hinder the translation of MGF extraction processes to industrial settings [13, 23]. In contrast, aqueous ethanol solutions have emerged as a compelling green alternative to organic solvent‐based systems. Their low toxicity, tunable polarity, renewable origin, and straightforward solvent recovery make them particularly advantageous for sustainable bioprocess development, as illustrated in Figure 1B [24, 25, 26, 27]. Despite these advantages, the combined effects of ethanol‐water ratio, temperature, and extraction time on the selective recovery of MGF from *M. indica* remain insufficiently explored within a rigorous statistical modeling framework. This knowledge gap is especially relevant considering that mango plant biomass represents an abundant and low‐cost agricultural residue with significant potential for valorization under circular bioeconomy principles. Although maceration is often regarded as a simple technique, its compatibility with aqueous ethanol solutions, low environmental footprint, and suitability for scale‐up make it an attractive platform for sustainable extraction processes when guided by advanced experimental design methodologies [19, 28, 29, 30].

A bibliometric analysis reinforces the need for methodological advancement in this area. A search in the Web of Science revealed 261 publications associated with the terms “mangiferin” and “extraction” since 1971, with a peak of 27 works published in 2022 (Figure 1C), demonstrating long‐standing scientific interest in isolating MGF. However, narrowing the search to “mangiferin” and “conventional extraction” resulted in only 24 publications (Figure 1D), indicating that few studies have systematically evaluated classical extraction techniques. More notably, only 10 publications were retrieved for “mangiferin” and “ethanol water” (Figure 1E), underscoring a significant scarcity of research dedicated to green, low‐toxicity, and scalable solvent systems. This discrepancy highlights a clear methodological gap: despite the recognized pharmacological importance of MGF, sustainable extraction strategies remain critically underexplored. These trends emphasize both the scientific relevance and the timeliness of investigating aqueous ethanol solutions for MGF recovery within a structured statistical framework.

In this context, the present study proposes an integrated and statistically guided strategy for the sustainable extraction of MGF from dried *M. indica* leaves using aqueous ethanolic maceration. The novelty of this work lies in the sequential integration of Taguchi screening and Box‐Behnken response surface optimization, enabling the identification of the most influential extraction variables followed by the refinement of their interaction and quadratic effects on MGF recovery. Unlike conventional extraction studies that focus primarily on maximizing extraction yield, the present work integrates process optimization with chromatographic and mass spectrometric confirmation of MGF, biochemical characterization, antioxidant profiling, and greenness assessment using the Analytical GREEnness (AGREE) metric approach. The optimized extracts were characterized in terms of total phenolic (TP) content, reducing sugars, protein concentration, and antioxidant capacity, including half‐maximal inhibitory concentration determination using the 2,2‐diphenyl‐1‐picrylhydrazyl (DPPH) radical scavenging assay. By integrating sustainable extraction, multivariate optimization, chemical identification, functional antioxidant evaluation, and analytical greenness assessment, this study provides a comprehensive, environmentally responsible, and potentially scalable framework for the recovery of MGF from an underutilized plant biomass. These findings contribute to the development of green bioprocesses and reinforce the potential application of MGF‐rich extracts in biopharmaceutical, nutraceutical, and cosmetic formulations.

## 2 Material and Methods

### 2.1 Materials

MGF standard (purity ≥ 98%, *M*_w_ ≈ 422.33 g/mol) and DPPH were obtained from Sigma‐Aldrich (St Louis, MO, USA). Mango samples were stored protected from light and moisture until used in the extraction experiments. Ethanol (purity of 99.8%) was purchased from Exodo Científica (Sumaré, SP, Brazil). All aqueous solutions were prepared using distilled water, and all additional reagents were of analytical grade and used without further purification.

### 2.2 Plant Material Preparation

*M. indica* L. plant biomass, consisting of leaves and bark from the Brazilian “Rosa” variety, was collected directly from trees located on the campus of the Lorena School of Engineering, University of São Paulo (EEL/USP), Brazil. The plant material was identified as *M. indica* L. var. “Rosa” based on its morphological characteristics and cultivation records from trees maintained on the EEL/USP campus. To minimize biological and environmental variability, all samples were harvested from the same tree, during the same season, and at the same time of day. The collection site was geographically referenced at 22°41′53.6″ S and 45°07′02.5″ W. The examined specimens have been properly preserved and deposited in the institutional reference collection of the USP, where they are currently undergoing the official accession and cataloguing process. Consequently, an official voucher specimen number has not yet been assigned at the time of manuscript submission. The voucher information will be made publicly available upon completion of the institutional cataloguing procedures. After collection, the leaves were manually inspected to remove damaged or unsuitable material. Selected leaves were washed with potable water to remove surface debris, followed by rinsing with distilled water to eliminate residual impurities. Excess moisture was removed by air drying, and the leaves were subsequently dehydrated in a laboratory oven (SolidSteel SSDicd, Piracicaba, SP, Brazil) at 50°C for 24 h. The dried material was ground using a high‐power blender (PH900 PR, 60 Hz, Philco, Joinville, SC, Brazil 60) to obtain the smallest particle size achievable under the operating conditions. The resulting powder was sieved through a 60‐mesh stainless steel sieve to ensure particle size uniformity, and its moisture content was subsequently determined. Finally, the processed material was stored in airtight containers at room temperature and protected from light until further analyses in order to preserve its physicochemical integrity [31].

### 2.3 Aqueous‐Ethanolic Maceration of MGF and Identification of Key Extraction Variables via Taguchi Design

The extraction technique and operating ranges were selected based on preliminary experiments and literature reports [32]. Preliminary experiments indicated that dried and ground mango leaves provided superior MGF extraction and were therefore selected for all subsequent experiments. Aqueous‐ethanolic maceration was chosen due to its simplicity, scalability, and compatibility with green solvent systems. The ranges of temperature, extraction time, agitation speed, and ethanol concentration were defined according to previous studies, laboratory feasibility, and solvent greenness.

For each experimental run, maceration was performed using a solid‐to‐liquid ratio of 1:40, in which 5 g of dried *M. indica* biomass (leaves) were mixed with 200 mL of an aqueous ethanol solution. The extraction conditions were established according to the Taguchi design, which served as a preliminary screening tool to identify the main variables influencing MGF recovery, namely temperature, extraction time, agitation speed, and ethanol concentration. To determine the relative contribution of each factor, a Taguchi design based on an L_9_ (3^4^) orthogonal array was applied following the methodology described by Yang and Tang [33]. This approach enables the simultaneous evaluation of multiple parameters while reducing the number of required experiments and maintaining statistical reliability. The four factors were assessed at three levels each: temperature (30, 40, and 50°C), extraction time (3, 7.5, and 12 h), agitation speed (100, 125, and 150 rpm), and ethanol concentration (20%, 50%, and 80% v/v). The nine resulting experimental combinations are presented in Table 1.

**TABLE 1:** Factors and levels evaluated in the Taguchi L_9_ (3^4^) experimental design applied to the extraction of mangiferin (MGF).

Assays	Temperature (°C)	Time (h)	Agitation speed (rpm)	[Ethanol] (%v/v)
**1**	30 (‐1)	3 (‐1)	100 (‐1)	20 (‐1)
**2**	30 (‐1)	7.5 (0)	125 (0)	50 (0)
**3**	30 (‐1)	12 (+1)	150 (+1)	80 (+1)
**4**	40 (0)	3 (‐1)	125 (0)	80 (+1)
**5**	40 (0)	7.5 (0)	150 (+1)	20 (‐1)
**6**	40 (0)	12 (+1)	100 (‐1)	50 (0)
**7**	50 (+1)	3 (‐1)	150 (+1)	50 (0)
**8**	50 (+1)	7.5 (0)	100 (‐1)	80 (+1)
**9**	50 (+1)	12 (+1)	125 (0)	20 (‐1)

*Values in parentheses represent the coded levels of each factor: ‐1, low level;0, intermediate level; and +1, high level.

Each extraction was performed in 250 mL Scotch flasks containing the dispersed biomass in the aqueous ethanol solution. The flasks were placed in a thermoregulated shaking water bath (Sppencer, model SP3065‐25), in which temperature and agitation were precisely controlled according to the Taguchi matrix. After maceration, the mixtures were vacuum filtered through a 125 µm pore size membrane to remove suspended solids and coarse particulates. The filtrates were subsequently centrifuged at 2500 × *g* for 15 min to eliminate residual fine particulates. The resulting supernatants were collected and stored at 8°C, protected from light, until further use [34]. MGF concentration in the extracts was quantified by ultraviolet‐visible (UV‐Vis) spectrophotometry, and data analysis was conducted using the signal‐to‐noise (S/N) ratio approach, which enabled the identification of the most favorable factor levels for maximizing extraction efficiency.

### 2.4 Optimization of Aqueous‐Ethanolic Maceration

The optimization of aqueous‐ethanolic maceration for MGF extraction was performed using a simultaneous multivariate approach based on a Box‐Behnken Design (BBD) implemented in Design‐Expert software (version 12.0). The experimental design consisted of fifteen runs, including three center points to assess model reproducibility and robustness. Three independent variables were investigated: extraction temperature, extraction time, and ethanol concentration in the aqueous ethanol solution, each evaluated at three levels (Table 2). This response surface methodology enabled the assessment of individual, interaction, and quadratic effects of the variables on MGF yield, allowing the identification of the optimal extraction conditions.

**TABLE 2:** Box‐Behnken experimental design evaluating the effects of temperature, time, and ethanol concentration on mangiferin (MGF) extraction.

Assays	Temperature (°C)	Time (h)	[Ethanol] (%v/v)
**1**	30	4	50
**2**	50	4	50
**3**	30	8	50
**4**	50	8	50
**5**	30	6	20
**6**	50	6	20
**7**	30	6	80
**8**	50	6	80
**9**	40	4	20
**10**	40	8	20
**11**	40	4	80
**12**	40	8	80
**13** *	40	6	50
**14** *	40	6	50
**15** *	40	6	50

* Conditions performed in the central points.

### 2.5 Quantification of MGF Concentration

The concentration of MGF in the extracts was determined by UV‐Vis spectrophotometry at 377 nm using a microplate reader (Multiskan Sky,Thermo Scientific, Waltham, MA, USA). Distilled water and ethanol were used as blank references, depending on the solvent composition of each sample. Absorbance values were quantified by correlation with a calibration curve constructed from standard MGF solutions prepared at concentrations ranging from 0 to 50 µg/mL.

### 2.6 Identification of MGF by Liquid Chromatography With Diode‐Array Detection and Electrospray Ionization Quadrupole Time‐of‐Flight Mass Spectrometry

High‐performance liquid chromatography with diode‐array detection (HPLC‐DAD) analyses were performed using a binary HPLC system (20AD Prominence, Shimadzu Co., Japan). Chromatographic separations were carried out on a Luna C18 column (250 × 3.0 mm, 5 µm; Phenomenex) maintained at 40°C, with a constant flow rate of 0.5 mL/min. Standard MGF solutions and extract samples were prepared at 1 mg/mL, and a 10 µL aliquot was injected for each run. The mobile phase consisted of Milli‐Q water (solvent A) and HPLC‐grade acetonitrile (solvent B), both acidified with 0.1% formic acid. Gradient elutions followed an adapted method from Ramirez et al. [35] 0–2 min at 20% B; 2–25 min at 50% B; 25–27 min ant 50% B (isocratic); 27–32 min at 100% B; 32–35 min at 100% B (isocratic); and returning to 20% B from 35 to 42 min for column re‐equilibration. UV detection was performed over a scan range of 190–400 nm. System control and data acquisition were carried out using LabSolutions (Shimadzu, version 3.60.361), and chromatographic data were processed in Origin 2024.

LC‐mass spectrometry (LC‐MS) analyses were performed on a high‐performance liquid chromatography (HPLC) system (20AD Prominence, Shimadzu Co., Japan) coupled to a Bruker MicrOTOF‐Q II MS. Separations were performed on a Luna C18 column (250 × 3.0 mm, 5 µm) with a 10 µL injection volume, using the same solvent system, gradient profile, flow rate (0.5 mL/min), and column temperature (40°C) described for the HPLC‐DAD method. Prior to MS analysis, eluates were monitored using a Shimadzu SPD‐20A PDA detector at 270 and 365 nm. The MS operated in positive electrospray ionization (ESI^+^) mode under the following conditions: drying gas temperature 200°C, gas flow 8 L/min, nebulizer pressure 4.0 bar, and capillary voltage 4.5 kV. Extract samples were analyzed by LC‐tandem MS (LC‐MS/MS) using total ion chromatograms and selected‐ion monitoring. For total ion chromatogram acquisition, the mass range was set from *m/z* 40 to 1000. MS/MS fragmentation was automatically triggered for the most intense ions to support compound identification.

### 2.7 Quantification of TPs, Proteins, and Reducing Sugars

The TP content of the extract samples was determined using the Folin‐Ciocalteu colorimetric method, as described by Singleton et al. [36], with adaptations for 96‐well microplates. In each well, 20 µL of the diluted extract was mixed with 100 µL of Folin‐Ciocalteu reagent (diluted 1:10) and 80 µL of sodium carbonate solution (7.5% w/v). The plate was incubated at 25°C for 60 min in the dark, and absorbance was measured at 765 nm using the same microplate spectrophotometer (described in the Section 2.5fic, USA). A calibration curve was prepared using gallic acid solutions ranging from 0 to 100 µg/mL, and results were expressed as milligrams of gallic acid equivalents per milliliter of extract (mg GAE/mL).

Protein concentration was quantified using the Bradford method with Coomassie Brilliant Blue G‐250, following the procedure described by Bradford [37]. For each assay, 160 µL of the diluted extract was combined with 40 µL of Bradford reagent in a 96‐well plate, in triplicate, yielding a final volume of 200 µL per well. After 5 min of reaction, absorbance was recorded at 595 nm using the same instr (ThermoScientific Multiskan Sky, USA). Protein concentration was calculated using a standard curve prepared with bovine serum albumin solutions ranging from 0 to 0.25 µg/µL.

Total reducing sugars were quantified using the dinitrosalicylic acid (DNS) method, following the procedures of Miller [38]. First, 1 mL of the diluted extract was mixed with 0.5 mL of DNS reagent in sealed test tubes. The mixtures were incubated in a boiling water bath (100°C) for 10 min to allow color development. After cooling to room temperature, 200 µL of each reaction mixture was transferred to a 96‐well microplate, and absorbance was measured at 540 nm using the same instrumenty, USA). All measurements were performed in triplicate, using distilled water as the blank. Quantification was based on a calibration curve prepared with glucose solutions ranging from 0 to 0.5 mg/mL.

### 2.8 Antioxidant Activity Evaluation

The antioxidant activity of the optimized extract samples was evaluated using the DPPH radical scavenging assay, following the methodology of Brand‐Williams et al. [39] with minor modifications. For each reaction, 1950 µL of a 0.1 mM DPPH solution in ethanol was mixed with 50 µL of the extract sample in black‐capped test tubes. The mixtures were homogenized for 1 min and incubated in the dark at 25°C for 30 min to allow the reaction to occur. After incubation, 200 µL of each reaction mixture was transferred to a 96‐well microplate, and absorbance was recorded at 515 nm using the same instrument (described in the *Section 2.5*). A control solution was prepared by replacing the extract with distilled water, and ethanol was used as the blank. The antioxidant capacity was expressed as the DPPH radical scavenging activity, calculated according to Equation (1):

(1) $$\begin{array}{ccc} & & \mathrm{DPPH}\;\mathrm{radical}\;\mathrm{scavenging}\;\mathrm{activity}\;(\%)\\ & & \;=\left[1\;-\;\left(\frac{\mathrm{AB}S_{\mathrm{sample}}}{\mathrm{AB}S_{\mathrm{control}}}\right)\;\times \;\;100\right] \end{array}$$

Additionally, the 50% inhibitory concentration (*IC*_50_) value (the concentration of extract required to decrease the initial DPPH radical concentration by 50%) was determined as an indicator of antioxidant potency.

### 2.9 Statistical Analysis

All experiments were conducted in triplicate (or duplicate when specified), and results were expressed as mean values ± standard deviation. The analysis of the experimental designs and the prediction of response variables were performed using Design‐Expert software (version 12.0). Response surface modeling and graphical visualization were performed in Statistica software (version 14.0.015) to interpret the individual and interactive effects of the independent variables. Statistical significance was set at *p* < 0.05.

### 2.10 AGREE Metric

To assess the environmental sustainability of the proposed analytical procedure, the open‐source AGREE Metric Approach and Software (AGREE, version 0.5 beta) tool was applied, integrating the twelve principles of green analytical chemistry (GAC), developed by Pena‐Pereira et al. [40]. The greenness profile of the method is based on an intuitive red‐yellow‐green color scale, which reflects the degree of compliance with each GAC principle.

## 3 Results and Discussion

### 3.1 Identification of Key Factors Influencing MGF Extraction by Aqueous‐Ethanolic Maceration

Preliminary analyses (data not shown) comparing different plant matrices demonstrated that mango leaves exhibited considerably higher MGF contents than bark samples. Given their superior yield, availability, and sustainability, the study proceeded exclusively with leaf‐derived extracts, reinforcing the suitability of this matrix as a renewable source of MGF.

**FIGURE 2:**
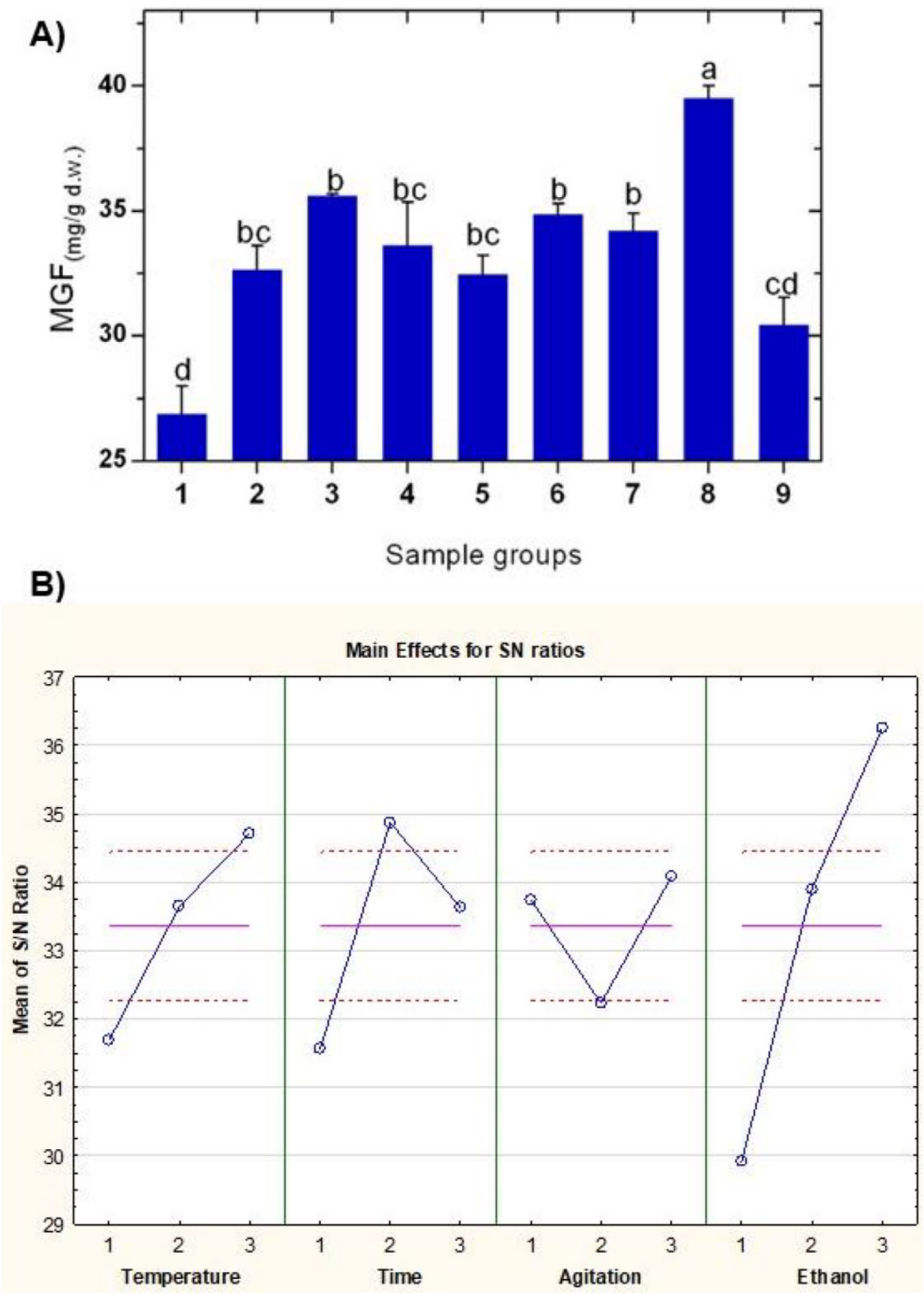
Mangiferin (MGF) extraction results obtained from the Taguchi experimental design (A). MGF yield (mg/g dry weight) for each of the nine experimental conditions. Run #8, performed under the parameters listed in Table 1, produced the highest MGF concentration. Different letters above the bars indicate statistically significant differences between treatments (*p* < 0.05). Main‐effects plots illustrate the influence of temperature, extraction time, agitation speed, and ethanol concentration on the signal‐to‐noise (S/N) ratio (B).

The Taguchi experimental design provided an efficient framework to systematically evaluate the effects of four extraction variables, temperature, extraction time, ethanol concentration, and agitation speed, on the recovery of MGF. Among these factors, temperature emerged as the most influential parameter, exerting a strong positive effect on MGF yield. As shown in Figure 2A, the highest extraction efficiency (39.87 ± 0.35 mg/g dry weight or d.w.) was obtained under the conditions of 50°C, 7.5 h, 100 rpm, and 80% ethanol (run #8, Table 1). This outcome is consistent with the well‐established role of temperature in improving solute solubility, solvent diffusivity, and mass‐transfer kinetics. However, excessively high temperatures may also promote thermal or oxidative degradation of phenolic compounds, including MGF, highlighting the need for moderate thermal input [41]. Previous studies have reported an optimal temperature window of 50 to 70°C for MGF extraction, where solubilization is maximized without compromising molecular stability [42].

Extraction time was also a significant contributor to process performance. The superior yields obtained at 7.5 h compared to 12 h suggest that prolonged maceration may lead to oxidative or thermal degradation of MGF and co‐extracted phenolics, reducing their availability and antioxidant activity. Long extraction times can favor reactions such as glycosidic bond cleavage, phenolic oxidation, or condensation between phenolics and other matrix components, all of which diminish the amount of intact MGF available in the extract [9]. The main‐effects plot in Figure 2B further supports the strong influence of both temperature and time, confirming their synergistic contribution to extraction efficiency.

In contrast, agitation speed showed no clear or consistent effect across the experimental runs, suggesting that its role may be secondary under the hydrodynamic conditions used. Because maceration is primarily governed by diffusion rather than convective transport, agitation often becomes relevant only when solvent penetration or boundary layer disruption is limiting. Under the conditions of this study, moderate temperatures and long contact times, agitation did not act as a determining factor in MGF release. Among solvent compositions, 80% (v/v) ethanol yielded the highest extraction efficiency, in agreement with findings from Anbalagan et al. [43] and other works on *M. indica* leaf extraction [12, 42, 44]. Hydroalcoholic mixtures are well known for their tunable polarity, allowing efficient solubilization of both moderately polar and hydrophilic phenolics. The Taguchi results confirm this principle, showing a clear positive trend with increasing ethanol content (Figure 2B). Solvents of intermediate polarity, such as aqueous ethanol, reduce the dielectric constant of the medium, enhancing the dissolution of C‐glucosylxanthones, flavonoids, and other antioxidant compounds [45].

However, polarity optimization is essential, as excessively high ethanol concentrations can reduce the extraction of certain hydrophilic phenolics or alter solute‐matrix interactions. For instance, Xu et al. [44] reported a decline in antioxidant performance at ethanol levels above 50%, attributed to changes in solubility equilibria or reduced extraction of polar co‐metabolites that may exert synergistic antioxidant effects. These findings underscore the importance of fine‐tuning solvent composition to balance maximum MGF recovery with the extraction of complementary bioactive constituents. This screening step provided essential insights that guided the subsequent response‐surface optimization through a BBD.

### 3.2 Box‐Behnken Optimization of MGF Extraction

Following the Taguchi screening, agitation speed was identified as a non‐significant factor and was excluded from subsequent optimization. Thus, the BBD focused on the three variables with the strongest influence on MGF extraction: temperature, extraction time, and ethanol concentration. The BBD consisted of fifteen experimental runs, including three central‐point replicates to assess model reproducibility and experimental variance. Compared to the maximum yield obtained in the Taguchi stage (39.87 mg/g d.w.), the BBD approach increased the recovery to 41.60–45.00 mg/g, demonstrating the additional value of modeling interaction and curvature effects. Whereas the Taguchi method captures only the main effects of each factor, BBD enables the evaluation of second‐order interactions and nonlinear contributions, thereby providing a more refined prediction of optimal extraction conditions.

A notable outcome of the BBD was the confirmation that minimal agitation combined with ethanol as a green solvent was sufficient to promote high extraction efficiency. The use of hydroalcoholic mixtures enhances the polarity balance of the medium, promoting solubilization of glycosylated xanthones such as MGF, while minimizing energy consumption compared to high‐shear or sonochemical methods. This aligns the proposed method with the principles of green chemistry and process intensification [46, 47, 48]. Across the experimental matrix (Table 3), MGF yield varied from 26.06 to 41.60 mg/g, demonstrating high sensitivity to the simultaneous adjustments of the three variables. The best‐performing conditions were observed in run #8 (50°C, 6 h, and 80% ethanol) and run #12 (40°C, 8 h, and 80% ethanol), confirming that ethanol concentration exerts the strongest positive effect, followed by extraction time and temperature. This behavior reflects classical solid‐liquid extraction principles, where increasing solvent strength enhances matrix penetration and reduces solute‐matrix interactions [49, 50].

**TABLE 3:** Box‐Behnken Design (BBD) showing the combinations of temperature, extraction time, and ethanol concentration tested for optimizing mangiferin (MGF) extraction, along with the corresponding yields (mg/g dry weight). Coded values for each factor are shown in parentheses.

Runs	Temperature (°C)	Time (h)	[Ethanol] (%v/v)	[MGF] mg/g d.w.
**1**	30 (‐1)	4 (‐1)	50 (0)	26.06
**2**	50 (+1)	4 (‐1)	50 (0)	36.27
**3**	30 (‐1)	8 (+1)	50 (0)	39.50
**4**	50 (+1)	8 (+1)	50 (0)	39.94
**5**	30 (‐1)	6 (0)	20 (‐1)	30.68
**6**	50 (+1)	6 (0)	20 (‐1)	34.06
**7**	30 (‐1)	6 (0)	80 (+1)	34.85
**8**	50 (+1)	6 (0)	80 (+1)	40.64
**9**	40 (0)	4 (‐1)	20 (‐1)	33.66
**10**	40 (0)	8 (+1)	20 (‐1)	37.77
**11**	40 (0)	4 (‐1)	80 (+1)	39.68
**12**	40 (0)	8 (+1)	80 (+1)	41.60
**13**	40 (0)	6 (0)	50 (0)	38.13
**14**	40 (0)	6 (0)	50 (0)	39.84
**15**	40 (0)	6 (0)	50 (0)	39.66

The reduced quadratic model derived from the BBD (Equation (2)) showed excellent predictive performance, with an *R* ^2^ of 0.91, indicating that over 91% of the variability in MGF recovery was explained by the combined linear, interaction, and quadratic effects of the selected variables. ANOVA demonstrated significant contributions from all three linear factors (temperature, time, and ethanol) as well as the interaction between temperature and time and the quadratic term of temperature (Table 4). The non‐significant lack‐of‐fit (*p* = 0.216) confirms model adequacy and reliability. The final reduced quadratic model, expressed in terms of coded factors and including the 95% confidence intervals of the regression coefficients, was described as follows:

(2) $$\begin{array}{ccc} {\text{[MGF]}}_{\text{(mg/g d .w.)}} & = & 6.21\;(\pm 0.12)+0.21\;(\pm 0.11)\;\times \;A+0.24\;(\pm 0.11)\\ & & \times \;B+0.21\;(\pm 0.11)\;\times \;C\;-0.22\;(\pm 0.15)\;\times \text{AB}\\ & & -\;0.28\;\left(\pm 0.16\right)\;\times \;A^{2} \end{array}$$

**TABLE 4:** Analysis of variance (ANOVA) results for the fitted quadratic model evaluating the significance of temperature, extraction time, ethanol concentration, and their interactions on mangiferin (MGF) yield. Significant terms (*p* < 0.05) indicate factors contributing meaningfully to the optimization model.

Source	Sum of squares	Degrees of freedom	Mean square	*F*‐value	*p*‐value	Significance
**Model**	1.72	5	0.3450	18.44	0.0002	Significant
**A – temperature**	0.3703	1	0.3703	19.79	0.0016	
**B – time**	0.4861	1	0.4861	25.98	0.0006	
**C – [ethanol]**	0.3633	1	0.3633	19.42	0.0017	
**AB**	0.1948	1	0.1948	10.41	0.0104	
**A^2^**	0.3105	1	0.3105	16.59	0.0028	
**Residual**	0.1684	9	0.0187			
**Lack of fit**	0.1571	7	0.0224	3.96	0.2164	Not significant
**Pure error**	0.0113	2	0.0057			
**Cor total**	1.89	14				
***R* ^2^ = 0.9111**						

**FIGURE 3:**
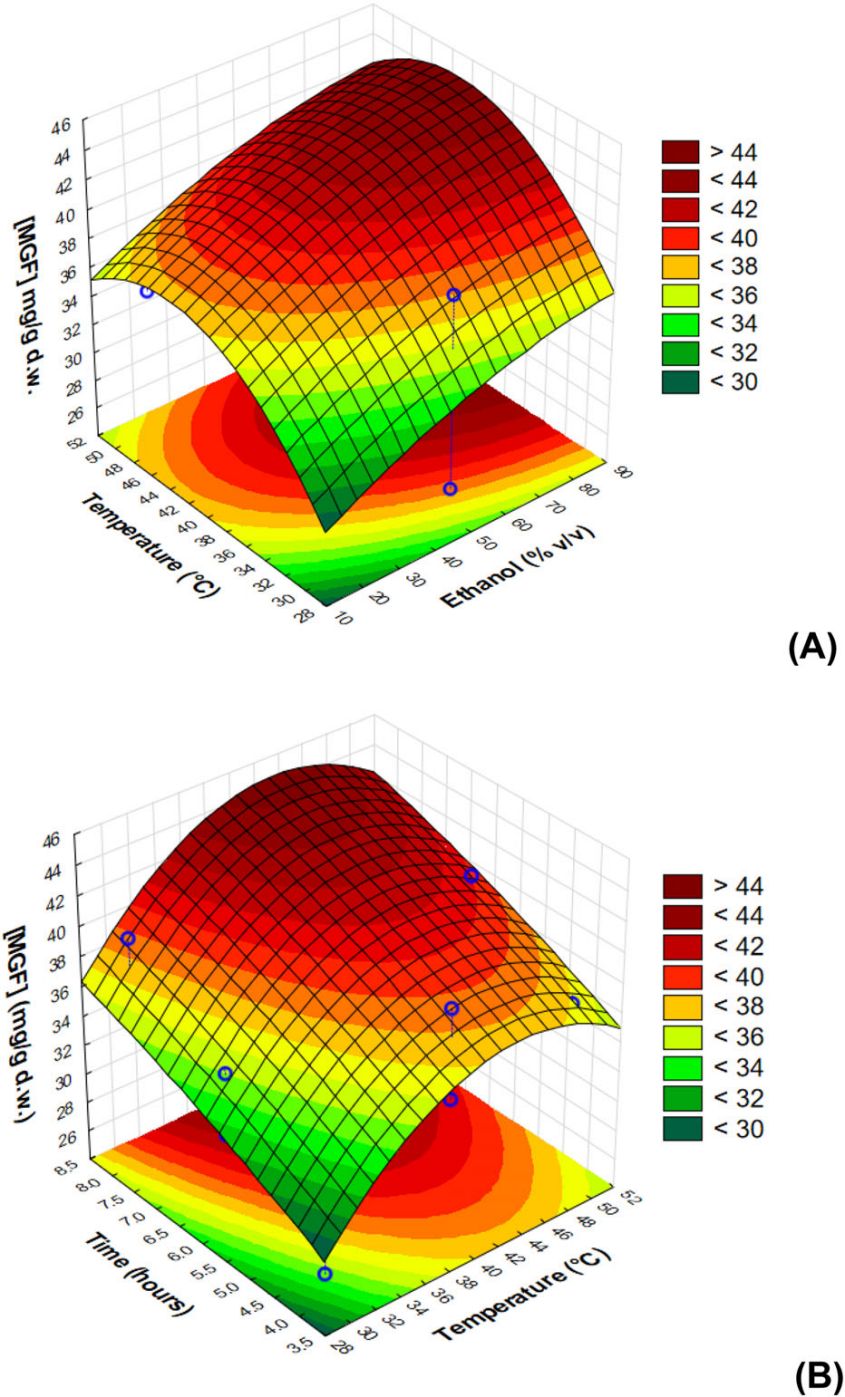
Response surface plots derived from the fitted quadratic model of the Box‐Behnken Design (BBD), showing the combined effects of process variables on mangiferin (MGF) extraction. Interaction between ethanol concentration and temperature (A), and between extraction time and temperature (B). Fixed factors for time in graph A and ethanol concentration in graph B were set according to the central point parameters (6 h and 50% v/v ethanol, respectively).

Surface response plots demonstrated that ethanol concentrations close to 80% (v/v) consistently maximized MGF extraction (Figure 3A). Temperature and time also showed strong synergistic effects: increasing temperature enhanced solubilization and diffusivity, while longer extraction times facilitated solvent penetration and the desorption of phenolic compounds from the plant matrix (Figure 3B). However, a plateau and, in some regions, a slight decline was observed at higher combinations of temperature and extraction time. This behavior suggests that prolonged exposure to thermal or oxidative conditions may promote partial degradation of MGF or competing phytochemicals within the extract. Using the numerical optimization module of Design Expert, the optimal extraction conditions were determined to be 39.9°C, 8 h, and 80% (v/v) ethanol, with a predicted yield of 44.50 mg/g d.w. within a 95% confidence interval (41.97–47.15 mg/g d.w.). Experimental validation under these optimized conditions resulted in an observed yield of 41.61 mg/g d.w., which fell within the predicted interval, confirming the robustness, accuracy, and predictive reliability of the fitted model. These optimized conditions were then used in all subsequent experiments.

In the study conducted by Zou et al. [42], MGF extraction from mango leaves was optimized using ultrasound‐assisted extraction (UAE), yielding 58.46 ± 1.27 mg/g under conditions of 44% ethanol, a 38:1 liquid‐to‐solid ratio, 60°C, and 200 W ultrasound power. While UAE achieves higher yields through acoustic cavitation and enhanced mass transfer, it requires specialized equipment, higher energy input, and precise temperature control. These operational demands increase cost and complexity and hinder scalability, as ultrasound propagation becomes less efficient in large extraction volumes and heterogeneous matrices. Additionally, sonication may generate localized heating and radical species, which can accelerate the degradation of thermolabile phenolics such as MGF [51, 52]. By contrast, the maceration‐based process optimized in this study operates under mild conditions, requires only simple and low‐cost equipment, and relies on a fully green ethanol‐water solvent system. Although the MGF yield (41.61 mg/g d.w.) is lower than that obtained with UAE, the optimized maceration approach offers superior accessibility, lower energy consumption, and greater scalability, making it a sustainable and industrially viable alternative aligned with green chemistry principles.

To further clarify the complementary roles of both experimental designs, a direct comparison between the Taguchi screening and the Box‐Behnken optimization is presented in Table 5. The Taguchi design enabled the rapid identification of the most influential extraction variables, whereas the BBD refined the process by modeling linear, interaction, and quadratic effects. Together, these approaches provided a more comprehensive optimization strategy and improved the predictive understanding of MGF recovery.

**TABLE 5:** Comparative summary of the Taguchi and Box‐Behnken experimental designs employed for mangiferin (MGF) extraction from *M. indica* biomass.

Aspect	Taguchi design	Box‐Behnken design
Primary objective	Screening of extraction variables	Optimization of selected variables
Variables evaluated	Temperature, extraction time, agitation speed, and ethanol concentration	Temperature, extraction time, and ethanol concentration
Statistical approach	Orthogonal array design	Response surface methodology
Experimental runs	Nine runs	Fifteen runs, including three central points
Model contribution	Evaluation of main effects	Evaluation of linear, interaction, and quadratic effects
Best MGF recovery	39.87 mg/g dry weight	41.61 mg/g dry weight (experimental) and 44.50 mg/g dry weight (predicted)

### 3.3 Identification of MGF and Quantitative Biochemical Profile of the Optimized *M. indica* Extract

The TP content of the optimized aqueous‐ethanolic extract was 4.36 ± 0.2 mg GAE/g d.w. (Table 6), a value consistent with works for *M. indica* leaves and reflecting the predominance of phenolic constituents in this plant matrix [53, 54]. HPLC‐DAD and HPLC‐ESI‐Quadrupole time‐of‐flight analyses confirmed the presence of MGF in the aqueous ethanolic extracts, with detailed chromatographic and mass spectrometric data provided in the Supporting Information (Figures S1–S3 and Table S1). Quantitatively, the extract displayed a chemical profile dominated by reducing sugars (73.12 mg/g d.w.), followed by MGF (41.60 mg/g d.w.), TP (4.36 mg GAE/g d.w.), and trace levels of soluble proteins (8.64 µg/g d.w.).

**TABLE 6:** Chemical composition and antioxidant activity of the optimized aqueous‐ethanolic extract from *M. indica* L. leaves.

Main components	Values obtained
**Total phenolics**	4.36 ± 0.2 mg GAE/g d.w.
**MGF**	41.60 mg/g d.w.
**Total proteins**	8.64 µg/g d.w.
**Total reducing sugars**	73.12 mg/g d.w.
**Inhibition of DPPH**	87.74% inhibition at 400 µg/mL

Abbreviations: DPPH, 2,2‐diphenyl‐1‐picrylhydrazyl; d.w., dry weight; GAE, gallic acid equivalents; MGF, mangiferin.

This composition reveals that reducing sugars represent 61.40% of the quantified constituents, MGF of 34.93%, TP of 3.66%, and soluble proteins only 0.007%. The disproportionate enrichment of MGF is especially notable: the MGF‐to‐TP ratio (MGF/TP of 9.54) demonstrates strong selectivity toward MGF under the optimized extraction conditions. This trend aligns with the chemical affinity of C‐glucosylxanthones for hydroalcoholic systems at elevated ethanol concentrations and reinforces MGF as the principal contributor to the extract's bioactive profile. The composition obtained here is consistent with literature reporting *M. indica* as a rich source of phenolic molecules, including gallic acid, ellagic acid, flavonoids, xanthones, and glycosylated tannins [55]. Pan et al. [56] also identified MGF (7.43%) as the main component and dominant phenolic compound in 70% ethanol extracts of mango leaves, supporting the trend observed in the present study. Notably, the TP content obtained here (4.36 mg GAE/g d.w.) exceeds values reported for young (3.49 mg GAE/g) and mature leaves (0.43 mg GAE/g) by Barreto et al. [57], suggesting that the extraction strategy applied was particularly effective for phenolic recovery. Variability in phenolic concentration across studies is well known and is influenced by genetic background, maturity stage, environmental conditions, and, most importantly, extraction methodology [58]. Although modern techniques such as pressurized liquid extraction can produce dramatically higher yields (854.7 mg/g d.w.) [59], these methods involve higher cost, energy demand, and operational complexity compared to the maceration approach employed here.

The protein content in the aqueous‐ethanolic extract of *M. indica* leaves obtained in this study was 0.007%. This low value aligns with reports that mango leaves generally contain minimal protein compared to other plant tissues, although levels may vary with tissue type and cultivar. In contrast, mango pulp exhibits substantially higher protein concentrations (0.6%) in Colombian varieties [60] and up to 1.5–5.5% in Peruvian cultivars. Similar trends have been observed in mangoes from other regions, with Java varieties containing 1–2% protein and Indian cultivars ranging from 0.5–1% [58, 61]. These comparisons clearly indicate that mango leaves possess markedly lower protein content than the pulp, reinforcing the expectation that proteins represent only a minor fraction of the leaf phytochemical composition.

In contrast to the low protein levels observed in this study, mango seeds contain 6–13% protein on a dry‐weight basis, depending on the cultivar [62]. Although moderate, this protein fraction is nutritionally valuable due to a favorable essential amino acid profile [63]. The markedly lower protein yield from mango leaves highlights their limited nutritional relevance in terms of protein, although they remain rich in bioactive compounds of pharmaceutical and nutraceutical interest. Protein composition may vary with leaf maturity, cultivar, and extraction conditions, as documented in previous studies [58, 64]. The optimized aqueous‐ethanolic extract contained 73.12 mg/g of reducing sugars, substantially higher than typically reported for mango leaves. The sugar‐to‐MGF ratio of 1.76:1 may reflect both intrinsic carbohydrate content and partial glucose release from glycosylated phenolics, including MGF itself. Compared with literature values, 2.72% in Langra and 1.90% in Chaunsa [65], the 61.40% observed here underscores the impact of raw‐material selection and extraction methodology. Pretreatments can further enhance sugar recovery. Harum Manis leaves yielded 415.02 mg/g fermentable sugars after acid‐alkaline pretreatment. Strong cultivar‐dependent variation has also been reported, with Karthakolomban showing the highest sugar content (0.305 mg/g) among five varieties [66].

### 3.4 Analysis of the Antioxidant Activity

**FIGURE 4:**
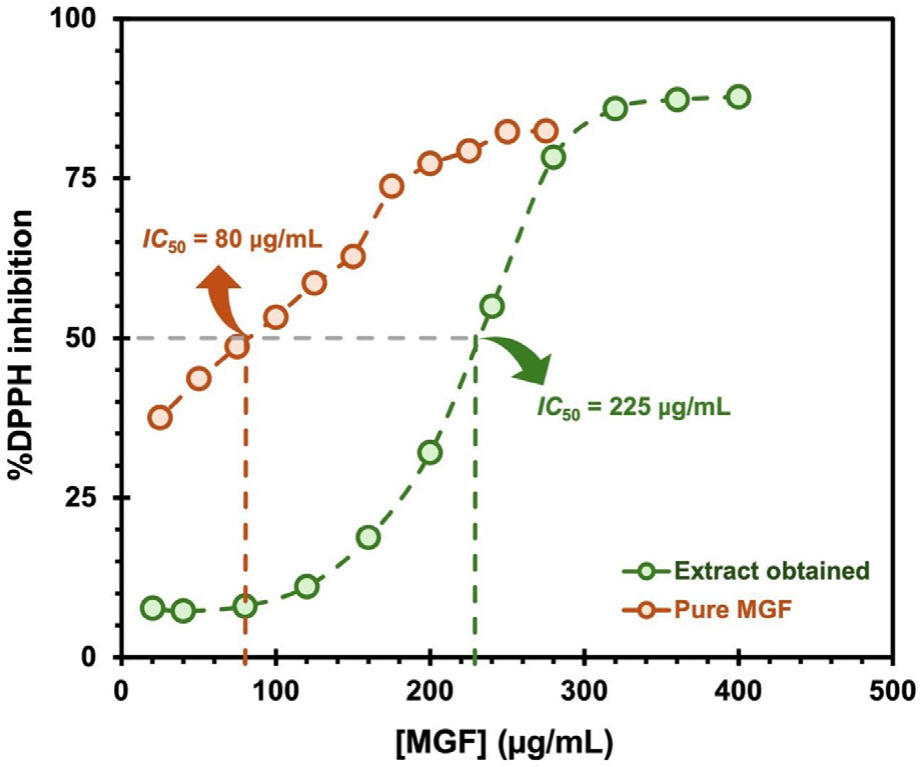
Determination of the *IC*_50_ value of the optimized aqueous‐ethanolic extract from *M. indica* leaves, calculated from the percentage of 2,2‐diphenyl‐1‐picrylhydrazyl (DPPH) radical inhibition.

The optimized aqueous‐ethanolic extract exhibited strong antioxidant capacity, achieving 87.74% DPPH inhibition at 400 µg/mL (Table 6 and Figure 4). The *IC*_50_ value of the extract was 225 µg/mL, whereas pure MGF presented an *IC*_50_ of 80 µg/mL, corresponding to a 2.8‐fold lower concentration required to reach 50% inhibition. However, because the extract represents a complex and non‐purified matrix, this comparison cannot be interpreted as a direct quantitative measure of the isolated antioxidant activity of MGF. Mango leaves contain several additional antioxidant constituents, including chlorophylls, traces of carotenoids, flavonoid glycosides, tannins, and minor xanthone derivatives, which may act synergistically with MGF. Therefore, any estimation of MGF's contribution should be considered indicative rather than definitive. The antioxidant response observed here reflects multicomponent interactions intrinsic to the phytochemical matrix rather than the exclusive action of a single compound [3, 67].

Purified MGF has been reported to achieve 94.2% DPPH inhibition at 200 µg/mL. The higher concentration required for comparable inhibition in the present extract is consistent with its compositional complexity and lower relative purity. Nevertheless, the substantial inhibitory effect confirms that the optimized extract constitutes a potent antioxidant source. At the molecular level, the antioxidant activity of MGF is associated with its polyaromatic xanthone scaffold, which enables resonance stabilization of phenoxy radicals through electron delocalization and efficient hydrogen atom donation [68]. Still, the high radical scavenging capacity observed cannot be attributed exclusively to MGF. Chlorophylls exhibit well‐documented radical quenching behavior mediated by conjugated porphyrin systems, while flavonoid glycosides and phenolic acids contribute complementary hydrogen‐donating and metal chelating mechanisms. Such compounds may enhance antioxidant performance through additive or synergistic effects, a phenomenon frequently observed in complex botanical matrices [69, 70].

Supporting the central role of phenolic constituents, Marcillo‐Parra et al. [71] demonstrated a strong positive correlation between TP content and antioxidant activity in mango by‐products, with MGF identified as the predominant phenolic compound. Variations in antioxidant response have also been linked to extraction methodology, since ultrasound‐assisted extraction generates distinct phenolic profiles and concentrations [72]. Moreover, beyond direct radical scavenging, MGF has been shown to activate the Nrf2 pathway, a key regulator of endogenous antioxidant defenses [73], indicating that its biological effects extend beyond simple chemical quenching mechanisms. Taken together, these findings confirm that the optimized aqueous ethanolic extract of *M. indica* leaves exhibits robust antioxidant activity. Although MGF plays a central role due to its abundance and structural reactivity, the contribution of other phytochemicals is likely significant. Future studies focusing on purification and fractionation will be essential to quantitatively delineate the specific contribution of MGF and to distinguish its isolated activity from the synergistic effects inherent to the extract matrix.

### 3.5 Greenness Assessment Profile

**FIGURE 5:**
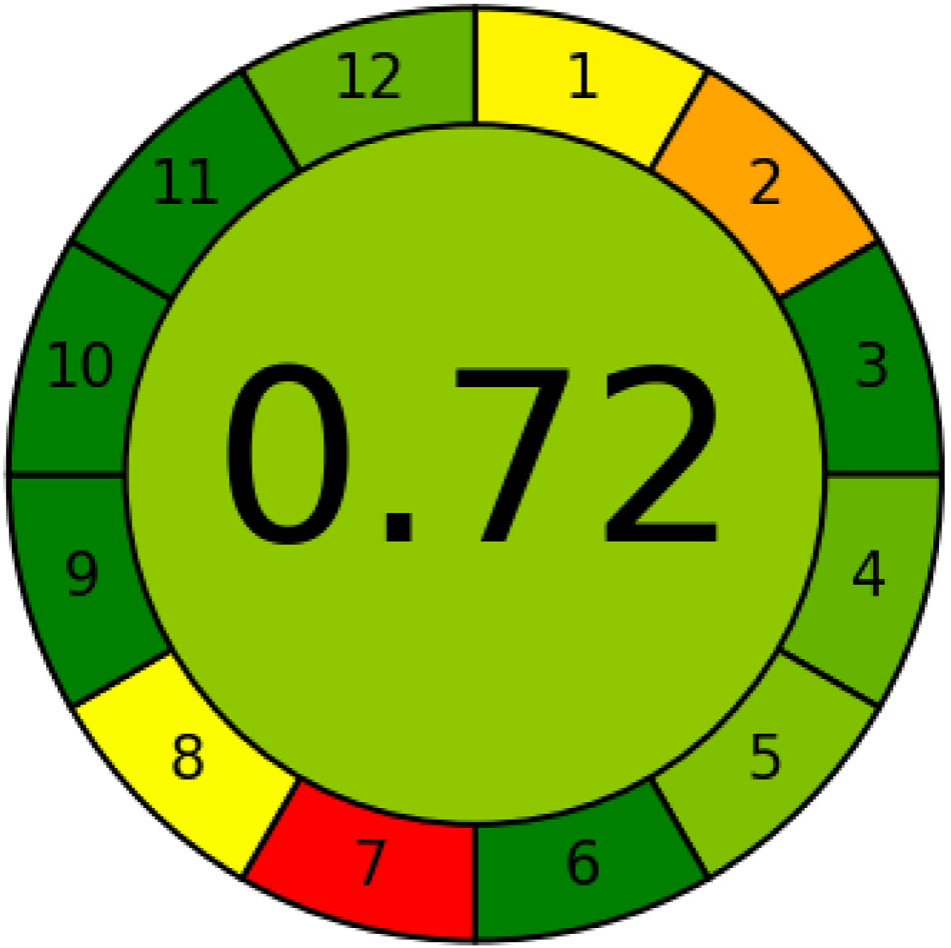
Analytical GREEnness (AGREE) pictogram obtained for aqueous‐ethanolic maceration as a mangiferin (MGF) extraction method applied to *M. indica* leaves, highlighting an overall greenness score generated.

The application of the AGREE metric to the aqueous‐ethanolic maceration process of *M. indica* leaves resulted in a global score of 0.72 (Figure 5), confirming its classification as an environmentally friendly method according to current green analytical benchmarks [74, 75]. This score is comparable to values reported by Soares et al. [74] for established extraction techniques assessed using the AGREE metric, including maceration (with 50% aqueous ethanol) at 20°C (0.77), subcritical water extraction (0.75), and microwave‐assisted extraction (0.74), and it exceeds the value observed for maceration at 60°C (0.68). These comparisons indicate that the proposed method operates within the upper range of green extraction platforms while maintaining methodological simplicity. The use of an ethanol‐water solvent system, moderate operating temperature (39.9°C), and conventional laboratory equipment contributed positively to the sustainability profile. Although the sample mass of approximately 5 g, solvent volume of approximately 250 mL, and extraction time of 8 h prevent the achievement of scores approaching unity, the obtained value demonstrates a balanced compromise between analytical performance and environmental impact. Notably, the score of 0.72 aligns with optimized spectrophotometric and chromatographic procedures reported in the literature (0.66–0.77), despite the present method involving lower instrumental complexity and reduced energy demand [40, 74, 76]. Overall, the greenness assessment reinforces that the optimized aqueous ethanol maceration constitutes a technically robust and environmentally responsible strategy for recovering bioactive compounds from plant biomass. Additional details are provided in Table S2 of the Supporting Information.

## 4 Conclusions

The combined application of Taguchi design and Box‐Behnken response surface methodology enabled a systematic and efficient optimization of the aqueous‐ethanolic maceration process for MGF extraction from *M. indica* leaves. The Taguchi screening established temperature, extraction time, and ethanol concentration as the most influential factors, providing a rational basis for further refinement. Using the BBD model, the optimal extraction conditions were defined as 39.9°C, 8 h, and 80% (v/v) ethanol, yielding 41.60 mg/g d.w. of MGF and confirming the predictive robustness of the statistical model. Chemical characterization of the optimized extract revealed a matrix enriched in MGF, reducing sugars, and phenolic compounds, with negligible protein content. The presence of MGF was confirmed by HPLC‐HRESI by the detection of its molecular ion peak. The extract also demonstrated strong antioxidant activity, reaching 87.74% DPPH inhibition at 400 µg/mL and exhibiting an *IC*_50_ of 225 µg/mL. These results highlight the intrinsic antioxidant capacity of MGF while also suggesting synergistic contributions from other phenolic and glycosylated constituents present in the extract. Overall, this study shows that aqueous‐ethanolic maceration, when guided by statistical optimization, constitutes a practical, energy‐efficient, and environmentally compatible strategy for MGF extraction. The high extraction yield, combined with the robust antioxidant potential of the resulting extract, reinforces its relevance for pharmaceutical, nutraceutical, and cosmetic applications. These findings establish a strong foundation for the continued valorization of *M. indica* leaves as a sustainable bioactive resource.

Subsequent studies should pursue the purification of MGF and assess the individual contributions of co‐extracted phenolics to the overall antioxidant response. The development of scalable purification strategies, such as aqueous biphasic systems or membrane‐based separations, will be crucial for industrial implementation. Additionally, incorporating the extract or purified MGF into delivery platforms (e.g., micelles, nanogels, and lipid carriers) may enhance stability and expand application potential. Biological evaluation in cell‐based or in vivo models will further clarify the therapeutic prospects of MGF‐rich systems and support their translation into functional and therapeutic products.

## Author Contributions

**Júlia Naelly Machado Silva**: methodology and writing – review and editing. **Astrid Corrales**: writing – original draft, methodology, investigation, and formal analysis. **André Moreni Lopes**: conceptualization, writing – review and editing, supervision, and project administration. **Massuo Jorge Kato**: writing – review and editing. **Felipe Rebello Lourenço**: writing – review and editing. **Vitor de Pádua Alves**: methodology, investigation, and writing – original draft.

## Conflicts of Interest

The authors declare no conflicts of interest.

## Supporting information




**Supporting File**: jssc70500‐sup‐0001‐SuppMat.docx.

## Data Availability

Data will be made available from the corresponding author on reasonable request.
