# Supplementary material for: Sustainable Extraction of Mangiferin From Mango Leaves Using Taguchi and Box‐Behnken Optimization: Biochemical Characterization and Antioxidant Profiling
Source: J Sep Sci. 2026 Aug 20;49(8):e70500. doi: 10.1002/jssc.70500 (PMC13492696; doi:10.1002/jssc.70500)
Supplement: Supplementary file 1 [file JSSC-49-e70500-s001.docx]

**Supplementary Material**

**Sustainable Extraction of Mangiferin from Mango Leaves Using**

**Taguchi and Box-Behnken Optimization:**

**Biochemical Characterization and Antioxidant Profiling**

**Astrid Corrales^1^, Vitor de Pádua Alves^2^, Júlia Naelly Machado Silva^3^,**

**Felipe Rebello Lourenço^4^, Massuo Jorge Kato^3^, André Moreni Lopes^1*^**

^1^Bioseparations and Nanoformulations Laboratory (BIOSEP NANOLAB), Department of Biotechnology, Lorena School of Engineering, University of São Paulo (DEBIQ, EEL/USP), Lorena, São Paulo, Brazil.

^2^Lorena Technical School (COTEL) - Prof. Nelson Pesciotta - Lorena School of Engineering, University of São Paulo, Lorena, São Paulo, Brazil.

^3^Natural Products Chemistry Laboratory, Institute of Chemistry, University of São Paulo (LQPN, IQ/USP), São Paulo, São Paulo, Brazil.

^4^Deparment of Faculty of Pharmaceutical Sciences, LabMEQ - Laboratory of Metrology, Examinology, and Quality by Design, University of São Paulo, São Paulo, Brazil.

**^*^Corresponding author:**

Prof. André M. Lopes

andreml@usp.br

Bioseparations and Nanoformulations Laboratory (BIOSEP NANOLAB)

Department of Biotechnology,

Lorena School of Engineering, University of São Paulo

The determination of MGF was performed using HPLC-DAD. A MGF standard and the extracts were initially analyzed to obtain the chromatographic profile and to characterize the UV absorption curves of the main constituents present (**Figure S1A**). And it was possible to confirm the presence of MGF in the ethanolic extracts of mango (**Figure S1B**) by analyzing the retention time and the UV absorption profile, which are consistent with the UV spectrum of the MGF standard.

For the extracts obtained from the leaves of varieties “Espada” and “Rosa”, chromatographic peaks corresponding to MGF were identified, exhibiting both the same retention time and the same UV absorption profile. In contrast, the ethanolic extract of variety “Haden” leaves showed a chromatographic peak with a slightly different retention time and a UV absorption profile that differed from that of MGF.

**Figure S1**. Overview of the chromatographic profiles obtained for the mangiferin (MGF) standard and for the different types of mango extracts, illustrating the distinct spectral characteristics and retention behaviors observed across matrices. Chromatogram of the mangiferin (MGF) standard obtained by HPLC-DAD with detection at 270 nm, together with its corresponding UV absorption spectrum (**A**). Overlay of HPLC-DAD chromatograms of mango leaf extracts from the “Espada”, “Haden”, and “Rosa” varieties recorded at 270 nm (**B**).

In the second step of the analyses, all samples were examined using HPLC-ESI-QTOF to validate the presence of MGF in ethanolic extracts, based on high-resolution mass spectrometry data and fragmentation pattern analysis. **Figure S2A** shows the fragmentation profile of the MGF standard in positive MS mode. **Figures S2B to S2D** present clear evidence of MGF in the leaf extracts through the identification of its characteristic fragmentation pattern.

 **(A)**

 **(B)**

 **(C)**

 **(D)**

**Figure S2.** High-resolution mass spectra confirming the presence of mangiferin (MGF) in mango leaf extracts based on the identification of its characteristic fragmentation profile. High-resolution mass spectrum of the MGF standard ([M+H]^+^ m/z 423.0932, 387.0718, 369.0615, 357.0612, 327.0498, 303.0500) (**A**). High-resolution mass spectra of leaf extracts from *M. indica* varieties “Espada” (**B**), “Haden” (**C**), and “Rosa” (**D**), confirming the presence of MGF based on the characteristic fragmentation pattern.

The data obtained by HPLC-DAD and HPLC-ESI-QTOF demonstrate the presence of MGF in all extracts, with the compound being the major constituent in the samples. No attempts were made to investigate the structural nature of the other constituents, including the substance that co-eluted with MGF in the extract from “Haden” mango leaves. Chromatograms of the samples by HPLC-DAD (**Figures S3A to S3C**).

**Figure S3**. HPLC-DAD chromatograms of leaf extracts from *M. indica* varieties “Espada” (**A**), “Haden” (**B**), and “Rosa” (**C**).

**Table S1.** Analytical workflow detailing experimental activities and their respective time requirements (in hours).

| **Activities** | **Time (h)** |
| --- | --- |
| Sample and equipment preparation | 2 |
| Data acquisition by HPLC-DAD | 6 |
| Data acquisition by HPLC-ESI-QTOF | 6 |
| Data analysis, processing, and report writing | 14 |
| Total | 28 |

**Table S2.** Green assessment of the optimized mangiferin (MGF) extraction method according to the 12 principles of green analytical chemistry (GAC) using the AGREE metric tool.

| Levels | Principles | Justification according to the extraction procedure |
| --- | --- | --- |
| 1 | Direct analytical techniques should be applied to avoid sample treatment | The method requires a hydroalcoholic extraction prior to analysis; therefore, direct analysis without sample preparation is not applicable. The procedure was classified as off-line analysis, which is common and acceptable for plant-based extraction methods. |
| 2 | Minimal sample size and minimal number of samples are goals | The method employed 10 g of plant material per extraction, which represents a moderate sample mass commonly used in phytochemical studies to ensure sufficient analyte recovery while maintaining analytical reliability. |
| 3 | In situ measurements should be performed when possible | The analytical measurements were carried out in the laboratory after sample extraction. In situ or on-line analysis was not feasible due to the solid nature of the matrix and the need for solvent-based extraction. |
| 4 | Integration of analytical processes and operations saves energy and reduces reagent consumption | The analytical workflow includes a limited number of essential steps (extraction, filtration, and occasional centrifugation), avoiding complex purification or multistage processing, which contributes to reduced operational complexity. |
| 5 | Automated and miniaturized methods should be selected | The procedure is manually operated and not miniaturized, relying on conventional laboratory equipment. While this limits automation and miniaturization, it enhances accessibility and reproducibility in standard analytical laboratories. |
| 6 | Derivatization should be avoided | No derivatization steps were required prior to analysis, preventing the use of additional reagents and avoiding the generation of unnecessary chemical waste. |
| 7 | Generation of large volumes of analytical waste should be avoided and properly managed | The extraction generates approximately 250 mL of waste per analysis; however, this waste consists of a benign ethanol-water mixture, which reduces the environmental impact compared to hazardous or halogenated solvents. |
| 8 | Multi-analyte or multi-parameter methods are preferred | The hydroalcoholic extract allows the determination of multiple analytes within a single extraction step, improving analytical efficiency and reducing the need for repeated sample preparation. |
| 9 | Energy consumption should be minimized | The extraction was primarily conducted under mild conditions, including room temperature during the final protocol, which contributes to reduced energy consumption. |
| 10 | Reagents from renewable sources should be preferred | Ethanol, a renewable solvent, was used as the main extraction solvent, combined with water, aligning with the preference for reagents derived from renewable resources. |
| 11 | Toxic reagents should be replaced with safer alternatives | The ethanol-water solvent system replaces more toxic organic solvents commonly used in extraction procedures, contributing to improved operator safety and reduced environmental risk. |
| 12 | Operator safety should be enhanced | The method avoids the use of highly toxic, volatile, or corrosive reagents, thereby minimizing health risks to the analyst and improving overall laboratory safety. |
